# From member creativity to team creativity? Team information elaboration as moderator of the additive and disjunctive models

**DOI:** 10.1371/journal.pone.0243289

**Published:** 2020-12-04

**Authors:** Yingjie Yuan, Daan van Knippenberg

**Affiliations:** 1 Faculty of Economics and Business, University of Groningen, Groningen, The Netherlands; 2 LeBow College of Business, Drexel University, Philadelphia, PA, United States of America; University of East Anglia, UNITED KINGDOM

## Abstract

One of the most fundamental questions in team creativity research is the relationship between individual member creativity and team creativity. The two answers that team creativity research has advanced–teams are more creative when their average member creativity is higher (the additive model) and teams are more creative when their most creative member is more creative (the disjunctive model) are straightforward. Surprising, however, is that neither the additive model nor the disjunctive model is consistently supported, begging the question of what moderates the predictive power of these models. We address this question by integrating individual-to-team creativity models with team process research. We propose that team information elaboration is a key moderating variable, such that average member creativity is more positively related to team creativity with higher information elaboration, and the highest member creativity is more positively related to team creativity with lower information elaboration. A multi-source study of 60 sales teams (483 employees) in a Chinese bakery chain supported these hypotheses. In addition, the study did not support the prediction that the most creative member’s outgoing advice ties (as a conduit for the dissemination of ideas) would further moderate the joint effect of the highest individual creativity and team information elaboration on team creativity.

## Introduction

To achieve and sustain business success, organizations increasingly adopt teamwork and build competitive advantage on the inimitable capital of creativity [1,2]–the generation of products, processes, and solutions to organizational problems that are both novel and useful [3–5]. This puts a premium on understanding the determinants of team creativity. One of the most fundamental questions in this respect is how individual members’ creativity contributes to team creativity. Creativity research has a longstanding tradition to see individual differences as a key determinant of creativity [6–8]. It is thus intuitive to focus on what individuals bring to the team [9–13]. The question of how individual member creativity relates to team creativity received surprisingly little research attention, however [14].

This modest attention is perhaps explained by the fact that it seems obvious that teams are more creative when their members are more creative. Indeed, along these lines team creativity research advanced two answers to the question how individual creativity is related to team creativity, and both received empirical support: the additive model [15] that posits that the sum or average (the latter in effect controls for team size) of member creativity is positively related to team creativity [16–18] and the disjunctive model [15] that posits that the creativity of the team’s most creative member is positively related to team creativity [16,19]. The support for the additive model and the disjunctive model is perhaps unsurprising, and the apparent obviousness of these relationships may explain why there is so little research on individual-to-team creativity. Importantly, however, and surprising from the perspective that support for the additive and disjunctive models is unsurprising, there are also empirical tests in which the additive model [20,21] and the disjunctive model [17,22] were not supported. The state of the science thus is such that both the additive model and the disjunctive model received mixed support, which suggests that the relationship of individual member creativity to team creativity is less straightforward and worthier of research effort than it may appear at first blush.

The mixed support for the additive and disjunctive models of individual-to-team creativity also suggests that it would be particularly valuable to consider moderation in the additive and the disjunctive models. The question on the research agenda thus is when average member creativity is more positively related to team creativity, and when highest member creativity is more positively related to team creativity. To address this question, we integrate individual-to-team creativity research with team process research in team creativity–two lines of research that so far developed in isolation from each other [14]. Team process research emphasizes how team creativity flows from synergetic processes in which team members collaborate to exchange and integrate information and perspectives [9,23,24] and thus indirectly is also affected by contextual factors like leadership and psychological climate that influence such synergetic processes [25–27]. Indeed, a recent review of the team creativity and innovation literature identified the synergetic process of team information elaboration (i.e., the exchange, discussion, and integration of task-relevant information and perspectives; [28]) as the core team process driving team creativity and innovation [14]. Our study builds on the recognition that team information elaboration is an additive process [29,30] to propose that team information elaboration moderates the predictive power of the additive and disjunctive models. Specifically, we propose that the additive model implies a team process in which members exchange and integrate creative contributions, and that therefore the additive model holds more with higher team information elaboration. Conversely, the disjunctive model implies a team process in which the team adopts the creative contributions of the most creative member rather than “diluting” these contributions by integration with other contributions. Therefore, the disjunctive model should hold more with lower information elaboration.

We further develop this analysis with a focus on how the most creative member’s creative contributions are disseminated throughout the team so that they can be adopted as team creative outputs (the additive process of team information elaboration implies that dissemination is inherent to the team process conducive to the predictive power of the additive model). Specifically, we propose that the most creative member’s outgoing advice ties are the conduits through which this member’s creative contributions are disseminated [31–33], and accordingly we expect that the disjunctive model is not just more predictive with lower information elaboration but also more so with higher centrality of the most creative member in the advice network.

In developing this moderation perspective on the additive and disjunctive models of individual-to-team creativity, the current study makes three contributions. First, we advance individual-to-team creativity research by developing a moderation model that helps understand the mixed support observed for the additive and disjunctive models. Second, we do so by integrating individual-to-team creativity research and team process research, two lines of research that by and large developed in isolation. Individual-to-team creativity research focused on how member creativity predicts team creativity without addressing the team process implied [34], whereas team process research has ignored how individual member creativity is core input in the creative team process [35], suggesting that both perspectives would benefit from integrating insights from the other perspective. Our study thus advances the theoretical integration in team creativity research by linking these two complementary perspectives. Third, team research more broadly has studied individual-to-team performance from the perspective of the Steiner (1972) [15] models (i.e., the origin of the additive and disjunctive models), and like team creativity research has done so in isolation from team process research. Moving beyond the traditional focus on the sum/average of member dispositions (i.e., the additive view), team researchers in recent years have shifted toward exploring the disjunctive influence of key players such as performance stars [36,37] and the most voicing member [38]. The benefits of information elaboration express themselves not only in creativity (and innovation), but more generally in knowledge work [28]. It is thus also worth studying the extent to which the current insights generalize to the team performance domain more broadly [39,40].

## Theory and hypotheses

Creativity in the workplace is defined as the generation of products, services, processes, and solutions to problems that are both novel and useful [3–5,41–44]. Team creativity thus refers to the generation of such novel and useful outcomes in teams. Different from the idea generation paradigm that stops at the generation of creative ideas in experimental research [45], the understanding of team creativity in organizations captures creative ideas that are put into actions to meet work challenges [14].

Creativity has long been understood to have a basis in individual differences—some individuals are more creative than others [7,41,44,46]. An obvious question to raise thus is how the individual creativity of team members affects team creativity. Even when researchers put the emphasis on synergetic team processes, and indeed the synergy that can be achieved in teamwork is sometimes seen as a key differentiator from individual creativity [5,47,48], the assumption would still be that team process is fueled by what individuals bring to the team. Teams rely on individual members to make creative contributions for teams to select, develop, and integrate into team outcomes [5,21,49,50]. Thus, given individual differences in creativity, it stands to reason to consider individual member creativity as a factor driving team creativity. As we noted in the introduction, at first blush it seems obvious that teams are more creative when their members are more creative. Suggesting there is more to the issue, however, team creativity research has advanced two different models of how individual member creativity is related to team creativity–the additive model and the disjunctive model–and both models received mixed support.

### From individual creativity to team creativity

The additive and disjunctive models are derived from Steiner’s (1972) [15] influential analysis of how individual contributions affect team performance. The model that is most often invoked in analyses of individual-to-team creativity is the additive model that posits that the sum or average of individual members’ creativity is positively related to team creativity–the creativity of all members contributes to team creativity [51–54], which does not negate individual differences in creativity, but sees less creative contributions too as adding to team creativity. Both operationalizations–average and sum–reflect the pooling of members’ creativity, but most empirical tests operationalized the additive model as the average rather than the sum of individual creativity to control for the impact of team size [22,55,56]. To align with prior studies as well as to maintain comparison in measurement with the disjunctive model on the same scale, from here on we therefore focus on the additive model in terms of average member creativity.

The disjunctive model sees member creativity contribute to team creativity through the creativity of the team’s most creative member. In the disjunctive model, the highest individual creativity is positively related to team creativity [16,17,57]. The disjunctive model obviously recognizes individual differences in creativity, and in contrast to the additive model sees less creative members not contribute to team creativity. By stressing the disproportionate contribution of the most creative member within a team, the disjunctive model is consistent with the growing recognition in team research that team members vary in their participation in and contribution to team processes [39,58]. This is not to say, however, that the disjunctive model negates the notion of teamwork. Teamwork can still be an integral part of delivering creative outcomes when the creativity of these outcomes is driven by the most creative member’s contributions. Other members may for instance contribute to the inspiration for, development of, and implementation of creative ideas through their knowledge and expertise and their efforts in trying out options in idea development. Indeed, examples of disjunctive team creativity abound. Pokémon Go, the most eye-catching mobile game in 2016, was a team product built upon the idea of designing an augmented reality game as envisioned by the team’s most creative member—John Hanke. Likewise, thousands of inventions from Nikola Tesla and his engineering teams were largely inspired by Tesla’s wild imagination.

As formulated by [15], the additive model and the disjunctive model do not specify the team process through which individual creative contributions result in team creativity. The models merely state how member creativity predicts team creativity. Possibly because the additive and disjunctive models do not specify team process, team creativity research has tested them as individual-to-team creativity models only and has paid little attention to the team process involved [9]. The issue is not that there is no attention to team process in team creativity research. There is, and research has quite consistently pointed to information elaboration (or related concepts or naming conventions such as knowledge integration and information exchange) as the key team process driving team creativity and innovation [14].

There is a well-developed understanding of teams as information processing systems [59,60]. In this perspective, teamwork is understood to revolve in important part around information integration, and the value of teamwork is seen as lying in the integration of member contributions (information, insights, ideas) into a team product (decisions, problem solutions, services, etc.). This perspective has also been applied to team creativity. The notion of team information elaboration has played a pivotal role in understanding team creativity, as captured in research on information sharing [61], information exchange [16,62], information elaboration [24,63,64], knowledge integration [65], and reflexivity [66,67]. We propose that in considering the team process implied by the additive and disjunctive models, we can integrate individual-to-team creativity research and team process research to identify information elaboration as a key moderator in the additive and disjunctive models.

### Team information elaboration as moderator in the additive model

To understand the boundary conditions of the additive and the disjunctive models, we have to develop our understanding of the process implications of both models. Such a consideration of team processes in effect invites an integration of the individual-to-team creativity perspective and the team process perspective in creativity. This is an approach that is consistent with [68] conclusion that a particular team process may be a moderator of the relationship between team input variables (e.g., team composition such as member creativity) and team outcomes (e.g., team creativity). In the specific case of our study, the issue thus is which team process would be involved in influencing the relationship between team member creativity and team creativity, and we propose that team information elaboration is the key process to consider here.

We first consider the role of elaboration in the additive model. We propose that the additive model in which team creativity is driven by the creativity of all members implies a process of information elaboration to integrate individual creative contributions (ideas, insights, further developments of others’ contributions) into team creative outcomes. This notion does not deny that some members are more creative than others, but it does imply that less creative contributions can add to more creative contributions to bring the team to higher levels of creativity. The issue here is not simply pooling contributions. This may be what happens in idea generation brainstorming-style [34,69], but in organizational practice, teams need to move beyond idea generation to further develop, select, and implement ideas. Moreover, while sometimes more ideas may be better, more typically teams can only implement one or a few ideas, and indeed may only need one idea (e.g., a solution to a job challenge). We see the additive model here not so much as speaking to the volume of contributions, but as revolving around the notions of synergy advanced in team creativity research: it is through the integration of different contributions from individual members that teams create new ideas, insights, and solutions that are different from the individual member contributions [5]. Team information elaboration is the process that captures this integration of individual ideas and insights [24].

We therefore conclude that the additive model of individual-to-team creativity implies a process of team information elaboration. Put differently, for the additive model to predict team creativity, teams need to integrate the contributions of different members. Capturing these notions in moderation terms, we propose that team information elaboration should be seen as a moderator in the additive model, such that average member creativity is more positively related to team creativity with higher team information elaboration.

#### Hypothesis 1

The average creativity of team members is more positively related to team creativity with higher team information elaboration.

### Team information elaboration as moderator in the disjunctive model

Where the additive model implies the integration of different creative contributions, the disjunctive model implies the opposite: it is the creative contribution of the most creative member undiluted by the creativity of others that drives team creativity. Accordingly, whereas we propose that the additive model is more predictive of team creativity with higher levels of team information elaboration, we may propose that the opposite holds for the disjunctive model. Information elaboration is exactly the process that would reduce the impact of the most creative member’s creativity on team creativity, because elaboration is associated with the integration of the contributions of different members [28], and thus with the generation of team outcomes that are not based on any particular member’s contribution. The integration of different perspectives in the process of information elaboration would transform the most creative members’ contributions through integration with other contributions. Information elaboration thus weakens the relationship between the most creative member’s creativity and team creativity, as team creative outputs are driven more by the integrative process of information elaboration and less by the most creative member’s contributions (i.e., this is not to say that information elaboration *must* involve the integration of different creative contributions; it is only to say that such integration is more likely the more teams engage in information elaboration).

Conversely, in situations of low information elaboration, teams may rely more on a process of adopting individual contributions, and this would foster a stronger link between the most creative member’s creativity and team creativity. Even when teams will not be perfect in recognizing creativity, in search of ways to meet business challenges they should be more likely to adopt more creative contributions. Teams in search of creativity also tend to be biased in favor of contributions of those having shown creative performance in the past [70], just as teams are more generally likely to prioritize the contributions of those with greater expertise (i.e., in the context of creativity, the most creative members; [71]). Adoption of the most creative member’s ideas may thus not only revolve around the greater creativity of this individual’s contributions, but also arise from greater attention to this individual’s contributions in matters requiring creativity. Accordingly, we may predict that the disjunctive model of individual-to-team creativity is more predictive of team creativity with lower information elaboration.

#### Hypothesis 2

The most creative member’s creativity is more positively related to team creativity with lower team information elaboration.

### Dissemination under low elaboration: Most creative member’s advice network centrality

Core to our analysis of moderation in the additive and disjunctive models are the process implications of these models in terms of team information elaboration, or the absence of it. There is one further issue to address in developing our analysis in full, however, and there is an asymmetry here in that it concerns the disjunctive model but not the additive model. For individual contributions to transform into a team outcome, the team needs to adopt a given initiative for implementation. A prerequisite for this adoption would be that the team is aware of the initiative. When the team engages in high levels of information elaboration, such awareness is inherent in the team process. When team members engage in an information elaboration process in which creative contributions are shared and integrated, whichever initiatives get adopted by the team in this process are by virtue of this shared interaction process adopted with the awareness of the members. When the team engages in little information elaboration, however, and conditions favor the disjunctive model, for the team to adopt the most creative member’s initiatives the team needs to be aware of these initiatives. Unlike for situations of high information elaboration, such awareness is not a given. When teams engage in little information elaboration, for team creativity to be driven by the most creative member’s creativity, the ideas of the most creative member need to be disseminated–spread within the team. Thus, whereas dissemination is inherent in the process of information elaboration that favors the additive model, we need to consider how the most creative member’s contributions get disseminated throughout the team under the low information elaboration conditions that favor the disjunctive model.

We argue that the predictive validity of the disjunctive model is not only contingent on team information elaboration, but also on the extent to which the most creative member’s creative contributions are disseminated under conditions of low information elaboration. The social network perspective is particularly useful in capturing the information flow from one individual to the rest of the team. Social network analysis focuses on the pattern of interpersonal relationships–ties–and is particularly suited to speak to how one individual is positioned to disseminate information to the rest of the team. In particular, prior network research has pointed out the importance to examine the strength of network ties in understanding the dissemination of complex information such as innovative ideas [72]. The most established way to capture the flow of work-related communication is through work-related advice ties [31,33,73–75]. Advice ties are interpersonal relationships characterized by the fact that the one individual in the relationship gives work-related advice to the other, where advice is broadly understood to include information, ideas, and insights.

Because the focus is on the flow of advice from one individual to the other, a distinction can be made between ties in which a focal individual receives (seeks) advice, and ties in which the focal individual gives advice. The fact that an individual gives advice does not imply that the individual receives advice or vice versa, just as the absence of advice-seeking does not imply the absence of advice-giving [32]. The act of giving advice does not only suggest the dissemination of information, ideas, and insights, but also indicates an influence on the recipient of the advice [33,76]. Thus, to capture the information flow and impact from the most creative member to the rest of the team, the appropriate focus is to look at this member’s outgoing advice-giving ties to the other members of the team. The more outgoing advice ties to other members of the team the most creative member has, the better this member is positioned to disseminate his or her creative contributions to the rest of the team, and to get these to be adopted by the team. This holds all the more because advice ties capture ongoing relationships in which the advisor role of providing information and advice over time adds to the credibility and legitimacy to influence advisees [32,77]. This notion of advice ties to the rest of the team is captured by the concept of network centrality [78,79]. A central position in the advice-giving network should help the most creative member disseminate creative initiatives to all or most team members.

We therefore propose that the predictive validity of the moderated disjunctive model further depends on the most creative member’s advice tie centrality. Under the conditions of low information elaboration that favor the disjunctive model, the most creative member’s creativity is more positively related to team creativity when this member possesses more central positions in advice networks. Under conditions of high information elaboration, such centrality is of less consequence because the team information elaboration process integrates and transforms contributions from all members rendering the disjunctive model less predictive.

#### Hypothesis 3

With lower information elaboration the relationship between the most creative member’s creativity and team creativity is more positive with higher advice centrality, whereas advice centrality has less moderating influence with higher information elaboration.

## Methods

### Data and sample

Sales teams of seventy-five bakery franchise stores from a company in the central part of China participated in this study. This company is known for novel pastry products and unique customer services in the bakery industry of China. It qualifies for our research question for two reasons: First, as stressed in the marketing and sales management literature, salespeople do not possess relevant KSAs or intra-firm influence to accomplish such tasks alone [80,81]. Organizations have increasingly organized their sales force in teams to handle the rapidly evolving demands of customers and competition [82,83]. Sales teams have to gather information from both clients and colleagues, develop and exploit such relations collectively in order to achieve long-term objectives [84]. In this way, contemporary salespeople are less like “lone wolves” and more like team players [82]. In the company from which our sample was drawn, we observed the same pattern. Salespeople are accountable for both team objectives and individual sales quotas. Members of a sales team work interdependently to decide and adjust sales strategies, and are seen as a team by themselves and others. For example, team members continuously learn sales skills from each other and actively coordinate their schedules, targets, and sales strategies when coping with complex sales situations. This allows us to investigate teamwork in the sales teams. In addition, the nature of franchised stores also guarantees homogeneous yet independent team settings for us to examine the research question.

Second, creative problem solving has been increasingly required for successful sales management in complex product-market situations. This trend has stimulated earlier studies of the creative performance of sales agents [49,85]. Salespeople must adopt novel ways to attract new customers and provide unique sales experiences. For instance, creative sales practice and services in sampled sales teams include retaining customer loyalty in social communication Apps, initiating DIY bakery activities in local orchards during holidays, and providing door-to-door delivery to student dorms in university parks. Management also encourages the creative culture among sales force by incorporating creativity measures in key performance indexes (KPIs) of sales teams, and rewards creative performance with promotion opportunities, yearly bonuses, and appraisals. Such a focus on creativity enables us to examine team creativity in these teams.

Notably, the focus on creativity in sales teams of a bakery chain implies a context of incremental creativity, where creative ideas for new sales practices would be often improvements and add-ons to ongoing practices rather than radical breakthroughs that would alter the existing business model or the current way it is done [86,87]. Our theory is agnostic as to whether this context of more incremental creativity as compared with radical creativity matters, but in contextualizing our research it may be useful to recognize that our findings derive from a field setting in which creativity emerging from the team would be incremental in nature.

In the course of two weeks, we sent paper-and-pencil surveys to all sales teams, including both team leaders (i.e., shop managers) and sales members. Team member surveys were administered on site in the first week. In the second week, all team leaders were invited to fill out the supervisor survey during their monthly review meeting at the corporate headquarter. Meanwhile, we obtained corporate assessments of team creativity from the HR office at the company’s headquarters. Follow-up reminders were sent to all absent employees and leaders.

567 out of 577 employees filled out the subordinate questionnaires, and 73 out of 75 team leaders filled out the supervisor counterparts. After matching corporate assessments of team creativity with supervisors’ ratings on subordinates’ creativity, we discarded seven teams due to the lack of corporate assessments, one team due to the lack of the team leader’s ratings, leaving 65 out of 75 teams—a valid response rate of 87 percent for the combination of three different data sources. Moreover, because a high response rate in each team network is required for accurate analyses at the network level, we further removed six teams with response rates of the network question below 80 percent, following suggestions in previous studies on team networks [74,88]. Thus, the final sample consisted of 486 employees from 61 teams of 3 to 21 members (*M*_*size*_ = 7.97, *SD*_*size*_ = 3.35).

This study was approved by the University of Groningen Research Ethics Committee from the Faculty of Economics and Business (Ref: FEB-20200429-11534). The process of data collection and analysis complied to the data protection laws and regulation of China. All subjects gave written informed consent when filling in the survey. The data were analyzed anonymously. The data that support the findings of this study are publicly available in the Center for Open Science (OSF) at https://osf.io/xz3jy/ (DOI 10.17605/OSF.IO/3ZEJC).

### Measures

#### Team creativity

We obtained KPI (Key Performance Indicator) scores for team creativity from the HR executive at the company’s headquarters, on a 100-point scale. This KPI system from which we elicited team creativity scores was tailored to meet strategic objectives of this company and has been tested for its validity and consistency before being put into practice. This KPI score of team creativity measures each team’s efforts and achievement against corporate strategies striving for customer-oriented and novel sales experiences. More specifically, this KPI score assesses to what extent creative ideas proposed by sales teams benefit the development of new sales strategies, the introduction of unique customer services, and the acquisition of new customers (i.e., in that sense, team creativity is understood here as ideas put into actions and not only as idea generation per se; [14]). These three component scores on novel sales strategies, unique customer services, and new customers carry equal weights and are added to a total score of up to 100 points. At the point of data collection, the company was not willing to provide us with the item-level breakdown that would enable us to compute Cronbach’s alpha for our sample. But in the later stage of data analysis, we were able to obtain a different sample of bakery sales teams (*N* = 106) from the same company based on the exact same measure for the analysis of inter-item reliability. This approach of reliability analysis was also used in prior studies to validate the reliability and validity of measurement instruments [89,90]. The Cronbach alpha here was 0.71, suggesting that the internal consistency of the creativity measure is acceptable.

As to the independence of this measure, this team creativity score was rated by performance management specialists who work independently from team operations and have received specific training in providing these ratings. Their ratings would then be checked and approved by performance managers before registered in the corporate eHR system. In that sense, these are not simply ratings from respondents untrained to rate creativity as mostly found in team creativity field research [14] but as close to expert assessments as we could come in this context.

A higher score indicates greater creativity in achieving the company’s performance objectives. In other words, even when they achieve the sales quota, teams that adopt little novelty to attract potential customers or generate new sales services would score low on this measure. For instance, ordinary ideas such as rewarding new customers or loyal customers with birthday coupons score lower than unique ideas like using WeChat to promote sales and retain customers or initiating DIY bakery activities to attract family customers. The measure thus fits the definition of creativity as the generation of something that is both novel and useful to meet business challenges [3,44].

#### Average individual creativity and the most creative member’s creativity

Team leaders assessed individual creativity of each subordinate using a 4-item scale [91] on a 10-point basis (1 = “Strongly disagree”, 10 = “Strongly agree”; *α* = .85). This scale has been translated into Chinese and validated in prior creativity research in China [92]. Sample items are, “this employee seeks new ideas and ways to solve problems”, and “this employee is a good role model for creativity”. Average individual creativity among team members therefore is operationalized as the mean of all individual creativity scores within a team. The most creative member of each team is identified as the individual with the highest creativity rating among members. The identification as the team’s most creative member thus reflects a purely relative assessment (i.e., in comparison with other team members) and does not rely on any absolute criteria for creativity. This operationalization allows the possibility of more than one member having the highest creativity score, but we expected this to be a low-frequency event. Indeed, in our sample, the vast majority of teams only had one member identified as most creative; one team had two members scoring the highest, and two teams have the same score for all members. We kept these teams in our analysis, because in practice some teams might have more than one most creative member. In our robustness tests, we found that dropping these teams would not alter the results of hypothesis analysis.

#### Team information elaboration

Team information elaboration was assessed with three items [29] on a 10-point scale (1 = “Strongly disagree”, 10 = “Strongly agree”; *α* = .81). A sample item is, “during the team task, we tried to use all available information”. We aggregated information elaboration to the team level. The inter-rater agreement and consistency indices validated our aggregation. ICC (1) was .55 (*F* [423, 848] = 4.69, BCa 95% CI = [0.50, 0.60]), and ICC (2) was .79 (*F* [423, 848] = 4.69, BCa 95% CI = [0.75, 0.82]). The *R*_*WG*_ statistic yielded a score of .81. According to LeBreton and Senter (2007) [93], ICC (1) scores above .40 imply a substantial effect size for grouping, and ICC (2) scores above .70 and *R*_*WG*_ indexes above .80 suggest high agreements among team members. Thus, our aggregation on mean ratings was justified.

#### Most creative member’s advice centrality

Because our research interest was to capture the extent to which intra-team network flows were conducive to the dissemination of the most creative member’s contributions, we employed the out-degree centrality score that reflects the amount of outgoing advice ties from a member to the rest within the advice-giving network to capture the flow of work-related information and influence from the most creative member to all the other members [73,94]. Although some prior studies operationalized the outgoing flow of advice as the incoming ties in advice-seeking networks (i.e., being sought out for advice) to avoid self-serving bias, we chose to directly measure the network of advice-giving ties for two reasons: seeking advice does not equate receiving advice [32], and more importantly not seeking advice cannot be equated with not receiving advice. Advice givers may proactively give unsolicited advice, which is especially relevant for new creative ideas that advice seekers may be unaware of. In other words, although measuring via advice-giving ties may be inflated by self-serving bias (i.e., it reflects well on the self to advice others), this is not an issue for our hypothesis testing because it only leads to more error variance which would make our findings more conservative.

Advice-giving ties were assessed via the roster method to improve recall [95,96]. Employees were requested to report “to what degree do you give this person professional advice when he/she has work-related problems?” on each team member listed on rosters, on a 6-point Likert scale (1 = “less often”, 2 = “several times a year”, 3 = “once a month”, 4 = “several times a month”, 5 = “several times a week”, 6 = “daily”). We computed the out-degree centrality in the social network analysis tool of UCINET. For the few teams where the highest creativity score was shared among more than one member, we used the mean of these members advice centrality scores as a conservative approach to keep these teams in the analysis.

#### Control variables

In preliminary analyses, we controlled a number of factors that prior studies reported as relevant [97,98], including team size, individual demographics (e.g., age, gender, and education), Big-5 personality traits, and employees’ organizational status (e.g., tenure and work shifts). Considering the power of our sample size and parsimonious modeling, we only included in final models control variables that related to team creativity significantly–team size.

## Results

Table 1 reports means, standard deviations, and correlations of variables in our regression models.

**Table 1:** Means, standard deviations, and correlations ^a^.

Variables	Mean	SD	1	2	3	4	5
1. Team creativity	59.02	10.71					
2. Team size	7.97	3.35	0.37**				
3. Average individual creativity	5.77	1.30	-0.14	-0.02			
4. Most creative member’s creativity	7.25	1.51	0.13	0.23***	0.67***		
5. Team information elaboration	7.48	0.64	0.11	0.01	0.05	0.04	
6. Most creative member’s advice centrality ^b^	0.91	0.16	0.01	0.09	-0.07	-0.10	0.07

^a^ *N* = 61.

^b^ *N* = 55.

* *p* < .05,

** *p* < .01,

*** *p* < .001.

We tested Hypothesis 1 and Hypothesis 2 in one hierarchical regression model, in order to examine the unique predictive power of each model controlling for the other (see Table 2). In a second regression analysis, we tested the three-way interaction posited in Hypothesis 3 (see Table 3).

**Table 2:** Moderating effects of information elaboration on the additive and the disjunctive models (Hypotheses 1&2) ^a^.

Variables	Step 1		Step 2		Step 3	
	*b (SE)*	*t*	*b (SE)*	*t*	*b (SE)*	*t*
Constant	59.02 (1.28)	46.04***	59.02 (1.27)	46.52***	59.06 (1.19)	49.69***
Team size	4.01 (1.29)	3.11**	3.30 (1.36)	2.43*	3.27 (1.28)	2.56*
Average individual creativity			-3.23 (1.77)	-1.83	-2.31 (1.71)	-1.35
Most creative member’s creativity			2.67 (1.82)	1.47	2.41 (1.72)	1.40
Team information elaboration			1.22 (1.28)	0.95	0.84 (1.21)	0.69
Average individual creativity × information elaboration					5.25 (1.93)	2.72**
Most creative member’s creativity × information elaboration					-6.75 (2.16)	-3.13**
*ΔR*^2^			.06*		.12**	
*R*^2^	.14**		.20*		.32***	

^a^ Depend Variable: team creativity. *N* = 61.

* *p* < .05,

** *p* < .01.

*Note*. To reduce unnecessary complexity and keep a parsimonious model, we dropped control variables that were found to have no relation with team creativity, including age (mean & diversity), gender (mean & diversity), tenure (mean & diversity), position (mean & diversity), work shift, educational background (mean & diversity) in teams.

**Table 3:** Regression results of the 3-way interaction of the disjunctive model (Hypotheses 3).

t	Step 1		Step 2		Step 3		Step 4	
	*b (SE)*	*t*	*b (SE)*	*t*	*b (SE)*	*t*	*b (SE)*	*t*
Constant	58.98 (1.29)	45.73***	59.61 (1.41)	42.29***	59.59 (1.33)	44.66***	59.95 (1.42)	42.37***
Team size	4.01 (1.30)	3.08**	2.90 (1.48)	1.96^†^	3.11 (1.39)	2.24*	2.96 (1.41)	2.11*
Average individual creativity	-1.32 (1.32)	-1.00	-2.68 (1.92)	-1.40	-1.78 (1.82)	-0.98	-1.89 (1.83)	-1.03
Team information elaboration	1.32 (1.31)	1.01	0.86 (1.51)	0.57	1.33 (1.47)	0.91	1.36 (1.48)	0.92
Average individual creativity × team information elaboration	0.79 (1.29)	0.61	0.36 (1.42)	0.25	5.50 (2.16)	2.55*	5.28 (2.18)	2.42*
Most creative member’s creativity			2.46 (1.93)	1.28	1.76 (1.91)	0.92	2.23 (2.01)	1.11
Most creative member’s advice centrality			-0.08 (1.43)	0.25	-0.60 (1.36)	-0.44	-1.70 (1.97)	-0.86
Most creative member’s creativity × team information elaboration					-6.78 (2.35)	-2.88**	-7.43 (2.50)	-3.00**
Most creative member’s creativity × most creative member’s advice centrality					1.91 (1.89)	1.01	0.91 (2.28)	0.40
Team information elaboration × most creative member’s advice centrality					-0.08 (1.28)	-0.06	0.42 (1.44)	0.30
Most creative member’s creativity × team information elaboration × most creative member’s advice centrality							2.26 (2.90)	0.78
*ΔR*^2^ (3-way interaction term)							.01	
*R*^2^	.18*		.15*		.31*		.32*	

^†^ *p* < .10,

* *p* < .05,

** *p* < .01.

**Fig 1:**
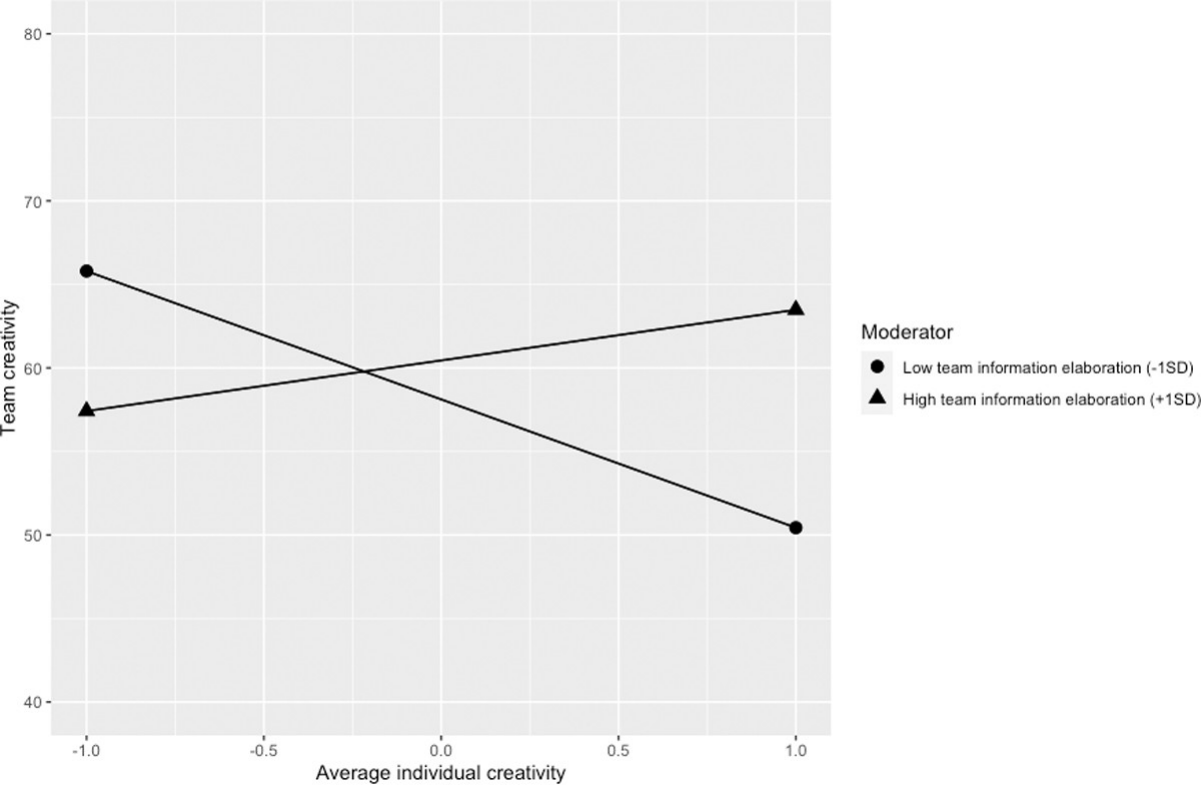
The additive model moderated by team information elaboration.

The entire regression model of moderated additive model and moderated disjunctive models explained 32% of the variance of team creativity, demonstrating the overall validity of predicting team creativity with individual creativity in different forms. Our findings revealed that team information elaboration positively moderated the impact of the additive model (i.e., average individual creativity) on team creativity (*b* = 5.25, *t* = 2.72, BCa 95% CI = [1.38, 9.13]), supporting Hypothesis 1 (see Fig 1). For a more accurate understanding of the moderating role of team information elaboration in Hypothesis 1, we employed the Johnson-Neyman technique to identify regions of significance using the R package of jtools [99]. Results showed that the relationship between average individual creativity and team creativity is significantly positive when team information elaboration is higher than 2.64 SD, and, surprisingly, significantly negative when team information elaboration is lower than -0.19 SD. Obviously, the finding that average member creativity is more positively related to team creativity with higher team information elaboration supports Hypothesis 1. We did not anticipate, however, that with lower information elaboration the relationship would turn negative. We will discuss the implication of this negative slope in the next section.

**Fig 2:**
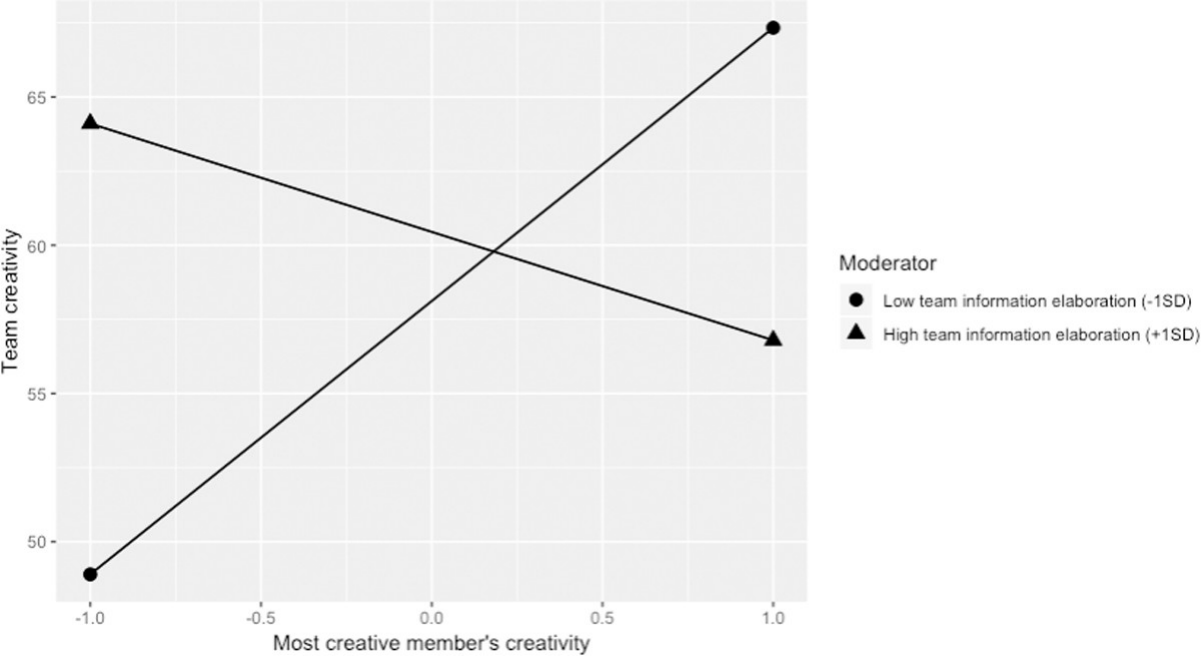
The disjunctive model moderated by team information elaboration.

For the disjunctive model, results showed that team information elaboration negative moderated the relationship between highest member creativity and team creativity (*b* = -6.75, *t* = -3.13, BCa 95% CI = [-11.08, -2.42], see Fig 2). The relationship between the most creative member’s creativity and team creativity is significantly positive when team information elaboration is lower than -0.16 SD, and significantly negative when team information elaboration is higher than 1.40 SD. The finding of a more positive relationship with lower information elaboration supports Hypothesis 2. As with our test of the additive model, however, we did not anticipate observing a negative relationship for higher elaboration. We will discuss the implication of this negative slope in the next section.

Hypothesis 3 proposed a three-way interaction of highest member creativity, team elaboration, and the most creative member’s advice network centrality. We found no support for this three-way interaction (*b* = 2.26, *t* = 0.78, BCa 95% CI = [-3.57, 8.09]; see Table 3).

## Discussion

Research of the relationship between member creativity and team creativity has been underappreciated, presumably because it seems so obvious that teams would be more creative when their members are more creative. As our conceptual and empirical analyses indicate, however, matters are less straightforward and worthier of study than they may appear at first blush. Research advanced two different models of how member creativity would affect team creativity and both have received mixed support. We address this issue by advancing moderated additive and disjunctive models of individual-to-team creativity. In identifying team information elaboration as a key moderator, we make a substantive step towards integrating individual-to-team creativity and team process research in team creativity. These findings, hypothesized as well as less anticipated, have some theoretical implications worth closer consideration.

### Theoretical implications

The theory and evidence we present for the moderated additive and disjunctive models address the issue of the mixed support for these models, which is a contribution in and of itself. The present study showed that the issue of individual-to-team creativity is not so much a “either-or” tradeoff between the additive and disjunctive models as previous research approached the issue [17], but rather under which conditions the additive model or the disjunctive model is more predictive. By putting this question on the agenda in combination with an answer supported by the evidence, our study breaks new ground for individual-to-team creativity research. In our analysis, we focused on what arguably is the core issue: the team process implied by the additive and disjunctive models. Team information elaboration is key here.

Hypothesis 1 and 2 were supported in that the additive model was more predictive of team creativity with higher information elaboration, and the disjunctive model was more predictive of team creativity with lower information elaboration. The additive and disjunctive models in their original forms as well as in the moderated forms we advanced imply that the relationship between average member creativity and team creativity, and highest member creativity and team creativity respectively, would be positive or, at most, range from positive to null. What we observed, however, is that with lower levels of information elaboration (-0.19 SD and below), the relationship between average creativity and team creativity was negative; and that with higher information elaboration (+1.40 SD and above), the relationship between highest member creativity and team creativity was negative. These findings were unanticipated, and at this point we can only propose a post hoc explanation for them.

One plausible explanation for these unexpected negative effects relates to how team members may respond to the team selection and integration of creative contributions. Low information elaboration implies that teams make a selection of creative contributions and drop the rest. When average individual creativity is high, many members will advance creative ideas but only a few get adopted when the team focuses on idea selection without much information elaboration. This may lead to negative responses, as the deference literature suggested that people are less likely to defer to others when they believe they themselves could provide more value to a group [100]. In contrast, teams with lower levels of average individual creativity might be less discouraged by the focus on idea selection with little information elaboration, for members do not have many great ideas to begin with. This might explain why we observed a negative effect of average creativity on team creativity at low levels of information elaboration. Representing the flip side of this argument, the most creative member of a team may be more appreciative of idea selection with little elaboration as this is likely to favor his or her contributions. In contrast, under conditions of high information elaboration, the most creative member may see his or her ideas “diluted” by the integration with less creative contributions. This may invite negative responses from the most creative member that may spark conflict that disrupts team performance [24,101–103]. This speculation can only be a post hoc explanation at this point, but it does suggest an intriguing line of future research to further develop the current moderation perspective on the additive and disjunctive models of individual-to-team creativity.

We did not find support for the moderating role of the most creative member’s advice centrality as proposed in Hypothesis 3. One explanation for this could be the choice of team networks we studied. Although advice networks are more specific than general team communication networks in capturing the diffusion of work-related ideas and opinions [74], it does not always refer to the influence of creative ideas. Anecdotal evidence implies that creative ideas at work sometimes spread out via more informal social networks (e.g., strong friendship ties). We would therefore be hesitant to dismiss the notion of dissemination through the social network as a moderating influence altogether, and speculate that this notion may hold in team networks that capture the influence of creative ideas more straightforwardly. The evidence of the importance of social networks in organizational behaviors, including creativity, is growing [44,50,95,96,104–106]. We encourage future studies to explore creativity-specific networks of teams and examine the dissemination logic of the disjunctive model in a more relevant context.

We examined the moderating role of team information elaboration in the present study. But it may not be the only (process) moderator involved. For instance, albeit the work teams in our sample have an interest in creative outcomes, teams do differ in their support for creativity and innovation [56,98,107]. This variation may further moderate the predictive validity of the additive and disjunctive model. In addition, research in team information elaboration has noted that the integration of different perspectives is an effortful process [28]. Teams are more likely to integrate a greater variance in contributions when members are more open-minded and motivated, as for instance captured by member need for cognition [108], member openness to experience [29], or member learning goal orientation [109]. These speculative examples illustrate that there is value-added in further developing the moderation perspective on the additive and disjunctive models of individual-to-team creativity. More broadly speaking then, a contribution of our study is to stimulate an integration of individual-to-team creativity and team process perspectives on team creativity that builds on and goes beyond the current integration.

We would also argue that the implications of the current study are not limited to team creativity research, but extend to team research more broadly. As noted by [110] and [111], when looking at the role of team member attributes there is a tendency in team research to apply the additive model by default, even when other individual-to-team models may be more valid, or should at least be considered. Moreover, Steiner’s (1972) [15] work has a strong influence in the consideration of individual-to-team models and in alerting researchers to alternatives for the additive model; but studies of individual-to-team performance have by and large limited themselves to “main effect” applications of these models [112–114], as per the state of the science in team creativity research). The current integrative perspective of moderated additive and disjunctive models may thus also inspire such integrative efforts in team research more broadly. The importance of information elaboration is not unique to team creativity and innovation and more broadly applies to knowledge work [28,59,60], and it is possible that information elaboration will emerge as an important moderator of the additive and disjunctive models also when considering other performance outcomes than creativity (e.g., decision making; [59]). It is also conceivable that there will be moderating influences that are specific to the performance outcome of interest, and the broader point here is not whether or not the current model applies beyond team creativity, but that the current theory and evidence suggests that team performance research more broadly may benefit from an integration of individual-to-team and team process models.

### Practical implications

Arguably, the evidence for the moderating role of team information elaboration makes the consideration of the additive model and the disjunctive model more actionable in practice. The previous focus on the additive model and the disjunctive model as main effects would suggest that organizations seeking creativity and innovation could either focus on putting teams of creative individuals together or identifying highly creative individuals to build a team around them that need not necessarily consist of otherwise creative individuals. Organizations only have so many degrees of freedom in composing teams, and to a large extent the question for practice is more how to manage the teams that one has than how to compose teams.

The nuance added by the moderated additive and disjunctive models suggests a different angle that we believe is helpful here. Although practical attempts to recruit creative individuals would still make sense, the current findings suggest that organizations can approach the issue more from a team process perspective. An important advice and precondition for success here would be, “know thy team”. Managers would need to have a sense of the creativity composition of their teams, specifically of the extent to which the average member is creative and the extent to which there is a member that stands out in terms of creativity. Contingent on the extent to which the manager is dealing with a team of creative members or a team with a highly creative member, the manager could aim to develop team processes to be conducive to the creative benefits of the team composition. For teams with creative members, the emphasis would be on information elaboration in shared efforts to generate creative outcomes; for teams with a highly creative member, the emphasis would be on supporting the most creative member in advancing and developing creative initiatives.

Importantly, managers are also advised to note the potential problems in processing creative members’ contributions. As we discussed above, the negative influence of average member creativity on team creativity at low levels of information elaboration, as well as the negative influence of highest member creativity at high levels of information elaboration, implies potential disruptions in the team process. When low information elaboration selectively adopts some members’ ideas but not the others, and when high information elaboration “dilutes" outstanding creative ideas with less creative ones, we speculate it might cause dissent (and even conflicts) among team members and in turn lower team creativity. We thus suggest managers pay attention to the consequences of processing member’s creative contributions, particularly in terms of members’ negative responses.

### Limitations and future directions

Despite its strengths, our study inevitably has limitations that future research may address to create a broader evidence base for our integration of perspectives. First, as a correlational study, the current study cannot speak to matters of causality. Team process is a challenge to manipulate experimentally with high validity, and our hypotheses required that teams could independently vary in their levels of information elaboration (and their social networks), which would be another challenge to implement experimentally. Given our hypotheses, the focus on a non-experimental field study may therefore be justified, but this does not change the fact that the current data cannot speak to causality and caution is in order interpreting correlational data.

We may also note that individual creativity was reported by team leaders rather than measured more objectively, or at least like our team creativity measure with trained external raters. Ideally, future research would rely on complementary evidence that is less subjective to bolster confidence in conclusions.

Another issue to note is that we tested our model in a context in which teams were designed for other purposes than creativity (i.e., sales teams), even when team creativity was included in the corporate KPI system and could clearly be seen as conducive to team performance. Moreover, this was a context focusing more on incremental creativity, where it was possible for individual team members to influence team creativity through individual creative contributions. Team contexts may differ along those lines, with some teams more explicitly charged with creativity (e.g., R&D teams) and presumably also an expectation for more radical creativity. Another contextual issue is that in our sampled teams, members had overlapping roles and similar expertise, which may have made it easier to let team creativity be driven by the creativity of just one member than in contexts where there is a need to combine the expertise of different members in tackling the job at hand. It is not clear how these factors affected our findings and would limit the generalizability to situations where the core charge is for the team to be creative, where more radical creativity is expected, or where there is a greater need to integrate the expertise of different members.

One could speculate that the need to integrate the expertise from different members would increase the push for information elaboration [115] and accordingly favor the additive model more than the disjunctive model. At the same time, we may note that the reliance on other’s expertise does not equate to reliance on other’s creativity; even when team success necessitates integrating contributions from different functional backgrounds, the *creativity* of team solutions may still be driven by one single member. There is also some evidence to suggest that the quality of single creative contributions matters more in radical than in incremental creativity [116]. There thus is insufficient basis for conclusions about the extent to which the current findings would generalize to other contexts with stronger creative demands or need to integrate different expertise, but this is clearly an issue worth exploring. As it is, a limitation of the current study is that it cannot speak to how much our findings generalize to setting with a stronger focus on radical creativity and integration of expertise. This is not to say that the context we studied is unusual. We contend that the creative context we studied is representative of lots of team contexts in organizations, where team creativity is in the service of overall team performance and team members’ roles sufficiently overlap to allow in principle any member to make a significant creative contribution to drive team creativity. Examples of such team contexts include teams of salespeople, academic researchers, and corporate board members.

In reference to the study context, we may also note that the teams in our study as locations of a bakery chain can and do function in relative isolation from the rest of the organization. We focused on team composition and internal team processes only, and in this context that may get to the core issues to consider (the variance explained by our models would seem to corroborate this impression). Teams in other organizations that are more firmly embedded in their organizational environment, may also find that their creativity is more affected by their external ties within the organization [117,118]. This is an issue that the current study does not speak to, but the evidence for the importance of external relations, for instance to generate support for the implementation of creative initiatives [119], is substantive enough to integrate this into further research concerning more embedded teams.

Another issue to note is the modest sample size of 61 teams in this study. It may raise the question of the statistical power of our findings. Following [120], we found the statistical power of our moderation effect to be 0.66 in a two-tailed *post hoc* power analysis test (*f*^*2*^ = 0.11, *α* = .05), which indicates a beta risk (Type ΙΙ error) of 34%. As suggested by [121], this is within the range of acceptable power sizes. Thus, we may infer that statistical power is not a major concern. That said, it would obviously be valuable to see future research replicate and extend the current findings to create more robust evidence.

Given that our study relied on a Chinese sample, one may wonder to what extent these findings generalize across cultures. In that respect, it is noteworthy that creativity research at both the individual level [122] and the team level [14] has not shown any cross-cultural difference in understanding the creative inputs and processes, and there is currently no basis to doubt the generalizability of Asian findings to “Western” settings and vice versa.

## Conclusion

The study of individual-to-team creativity has been underappreciated, presumably because the first-blush notion that teams are more creative when their members are more creative seems too obvious to warrant serious research attention. As shown in our study, however, the issue is more complex and by implication worthier of study: team information elaboration moderates the extent to which team creativity is driven by the average creativity of members or by the most creative member’s creativity. These insights do not just advance individual-to-team creativity research, but also contribute to an integration of individual-to-team creativity research and team process research. This integration has implications for team research beyond the study of team creativity, and may thus broadly inspire further development of this integrative approach in team research.

## References

[1] Wuchty S, Jones BF, Uzzi B. The increasing dominance of teams in production of knowledge. Science (80-). 2007;316: 1036–1039. 10.1126/science.1136099 17431139

[2] Prabhu V, Sutton C, Sauser W. Creativity and Certain Personality Traits: Understanding the Mediating Effect of Intrinsic Motivation. Creat Res J. 2008;20: 53–66.

[3] Amabile TM. A Model of Creativity and Innovation in Organizations. Res Organ Behav. 1988;10: 123.

[4] Shalley CE, Zhou J. Organizational creativity research: A historical overview BT—Handbook of organizational creativity. Erlbaum; 2008 pp. 3–31.

[5] Hargadon AB, Bechky BA. When collections of creatives become creative collectives: A field study of problem solving at work. Organ Sci. 2006;17: 484–500.

[6] Amabile TM. From individual creativity to organizational innovation. 1988.

[7] van Knippenberg D, Hirst G. Cross-level perspective on creativity at work: Person-in-situation interactions BT—Oxford handbook of creativity, innovation, and entrepreneurship. Oxford University Press; 2015.

[8] Zhou J, Hoever IJ. Research on Workplace Creativity: A Review and Redirection. Annu Rev Organ Psychol Organ Behav. 2014;1: 333–359.

[9] Taggar S. Individual creativity and group ability to utilize individual creative resources: A multilevel model. Acad Manag J. 2002;45: 16–330.

[10] Gilson LL, Shalley CE. A little creativity goes a long way: An examination of teams’ engagement in creative processes. J Manage. 2004;30: 453–470.

[11] Miron-Spektor E, Erez M, Naveh E. Balancing Innovation Attention-To-Detail and Outcome-Orientation to Enhance Innovative Performance. Acad Manag Proc. 2007;2007: 1–7.

[12] Miron-Spektor E, Erez M, Naveh E. Effect of Conformist and Attentive-To-Detail Members on Team Innovation: Reconciling the Innovation Paradox. Acad Manag J. 2011;54: 740–760.

[13] Bissola R, Imperatori B, Colonel RT. Enhancing the creative performance of new product teams: An organizational configurational approach. J Prod Innov Manag. 2014;31: 375–391.

[14] van Knippenberg D. Team Innovation. Annu Rev Organ Psychol Organ Behav. 2017;4: 211–233.

[15] Steiner DI. Group processes and group productivity. Academic; 1972.

[16] Gong Y, Kim TY, Lee DR, Zhu J. A multilevel model of team goal orientation, information exchange, and creativity. Acad Manag J. 2013;56: 827–851.

[17] Taggar S. Group composition, creative synergy, and group performance. J Creat Behav. 2001;35: 261–286.

[18] Navaresse DO, Yauch CA, Goff K, Fonseca DJ. Assessing the Effects of Organizational Culture, Rewards, and Individual Creativity on Technical Workgroup Performance. Creat Res J. 2014;26: 439–455.

[19] Triandis HC, Bass AR, Ewen RB, Mikesell EH. Team creativity as a function of the creativity of the members. J Appl Psychol. 1963;47: 104–110.

[20] Hanke RCM. Team creativity: A process model. ProQuest Dissertations and Theses. 2006.

[21] Mathisen GE, Martinsen O, Einarsen S. The relationship between creative personality composition, innovative team climate, and team innovativeness: An input-process-output perspective. J Creat Behav. 2008;42: 13–31.

[22] Kurtzberg TR. Creative styles and teamwork: Effects of coordination and conflict on group outcomes. ProQuest Dissertations and Theses. 2000.

[23] Harvey S, Kou C-YY. Collective Engagement in Creative Tasks: The Role of Evaluation in the Creative Process in Groups. Adm Sci Q. 2013;58: 346–386.

[24] Hoever IJ, van Knippenberg D, van Ginkel WP, Barkema HG. Fostering Team Creativity: Perspective Taking as Key to Unlocking Diversity’s Potential. J Appl Psychol. 2012;97: 982–996. 10.1037/a0029159 22774764

[25] Goncalo JA, Duguid MM. Follow the crowd in a new direction: When conformity pressure facilitates group creativity (and when it does not). Organ Behav Hum Decis Process. 2012;118: 14–23.

[26] Goncalo JA, Chatman JA, Duguid MM, Kennedy JA. Creativity from Constraint? How the Political Correctness Norm Influences Creativity in Mixed-sex Work Groups. Adm Sci Q. 2015;60: 1–30.

[27] Jia L, Shaw JD, Tsui AS, Park T-Y. A social-structural perspective on employee-organization relationships and team creativity. Acad Manag J. 2014;57: 869–891.

[28] van Knippenberg D, De Dreu CKW, Homan AC. Work group diversity and group performance: An integrative model and research agenda. J Appl Psychol. 2004;89: 1008–1022. 10.1037/0021-9010.89.6.1008 15584838

[29] Homan AC, Hollenbeck JR, Humphrey SE, van Knippenberg D, Ilgen DR, van Kleef GA. Facing Differences With an Open Mind: Openness to Experience, Salience of Intragroup Differences, and Performance of Diverse Work Groups. Acad Manag J. 2008;51: 1204–1222.

[30] van Knippenberg D, Kooij-de Bode HJM, van Ginkel WP. The Interactive Effects of Mood and Trait Negative Affect in Group Decision Making. Organ Sci. 2010;21: 731–744.

[31] Ibarra H, Andrews SB. Power, Social Influence, and Sense Making: Effects of Network Centrality and Proxinnity on Employee Perceptions. Adm Sci Q. 1993;38: 277–303.

[32] Agneessens F, Wittek R. Where do intra-organizational advice relations come from? The role of informal status and social capital in social exchange. Soc Networks. 2012;34: 333–345.

[33] Brass DJ. Being in the Right Place: A Structural Analysis of Individual Influence in an Organization. Adm Sci Q. 1984;29: 518–539.

[34] Pirola-Merlo A, Mann L. Relationship between individual creativity and team creativity: Aggregating across people and time. J Organ Behav. 2004;25: 235–257.

[35] Yuan F, Zhou J. Effects of cultural power distance on group creativity and individual group member creativity. J Organ Behav. 2015;36: 990–1007.

[36] Kehoe RR, Lepak DP, Bentley FS. Let’s Call a Star a Star: Task Performance, External Status, and Exceptional Contributors in Organizations. J Manage. 2018;44: 1848–1872. 10.1177/0149206316628644

[37] Call ML, Nyberg AJ, Thatcher SMB. Stargazing: An integrative conceptual review, theoretical reconciliation, and extension for star employee research. J Appl Psychol. 2015;100: 623–640. 10.1037/a0039100 25822068

[38] Li N, Zhao HH, Walter SL, Zhang X-A, Yu J. Achieving More With Less: Extra Milers’ Behavioral Influences in Teams. J Appl Psychol. 2015;100: 1025–1039. 10.1037/apl0000010 25664471

[39] Humphrey SE, Aime F. Team Microdynamics: Toward an Organizing Approach to Teamwork. Acad Manag Ann. 2014;8: 443–503.

[40] Mathieu JE, Tannenbaum SI, Donsbach JS, Alliger GM. A review and integration of team composition models: Moving toward a dynamic and temporal framework. J Manage. 2014;40: 130–160.

[41] Amabile TM. The social psychology of creativity: A componential conceptualization. J Pers Soc Psychol. 1983;45: 357–376.

[42] Woodman RW, Sawyer JE, Griffin RW. Toward a theory of organizational creativity. Acad Manag Rev. 1993;18: 293–321.

[43] Oldham GR, Cummings A. Employee creativity: Personal and contextual factors at work. Acad Manag J. 1996;39: 607–634.

[44] Perry-Smith JE, Shalley CE. The social side of creativity: A static and dynamic social network perspective. Acad Manag Rev. 2003;28: 89–106.

[45] Simonton DK. Scientific Creativity as Constrained Stochastic Behavior: The Integration of Product, Person, and Process Perspectives. Psychol Bull. 2003;129: 475–494. 10.1037/0033-2909.129.4.475 12848217

[46] Staw BM. Is Group Creativity Really An Oxymoron?: Some Thoughts on Bridging the Cohesion?Creativity Divide. Res Manag Groups Teams. 2009;12: 311–323.

[47] Kurtzberg TR, Amabile TM. From Guilford to Creative Synergy: Opening the Black Box of Team-Level Creativity. Creat Res J. 2001;13: 285–294.

[48] Harvey S. Creative synthesis: Exploring the process of extraordinary group creativity. Acad Manag Rev. 2014;39: 324–343.

[49] Sung SY, Choi JN. Effects of team knowledge management on the creativity and financial performance of organizational teams. Organ Behav Hum Decis Process. 2012;118: 4–13.

[50] Perry-Smith JE, Shalley CE. A Social Composition View of Team Creativity: The Role of Member Nationality-Heterogeneous Ties Outside of the Team. Organ Sci. 2014;25: 1434–1452.

[51] Shin SJ, Zhou J. When is educational specialization heterogeneity related to creativity in research and development teams? Transformational leadership as a moderator. J Appl Psychol. 2007;92: 1709–1721. 10.1037/0021-9010.92.6.1709 18020807

[52] van Kleef GA, Anastasopoulou C, Nijstad BA. Can expressions of anger enhance creativity? A test of the emotions as social information (EASI) model. J Exp Soc Psychol. 2010;46: 1042–1048.

[53] Tsai WC, Chi NW, Grandey AA, Fung SC. Positive group affective tone and team creativity: Negative group affective tone and team trust as boundary conditions. J Organ Behav. 2012;33: 638–656.

[54] Drazin R, Glynn MA, Kazanjian RK. Multilevel Theorizing about Creativity in Organizations: A Sensemaking Perspective. Acad Manag Rev. 1999;24: 286–307.

[55] Chen G, Farh J, Campbell-Bush EM, Wu Z, Wu X. Teams as Innovative Systems: Multilevel Motivational Antecedents of Innovation in R&D Teams. J Appl Psychol. 2013;98: 1018–1027. 10.1037/a0032663 23565898

[56] West MA, Anderson NR. Innovation in top management teams. J Appl Psychol. 1996;81: 680–693.

[57] Sacramento CA, Dawson JF, West MA. Team creativity: more than the sum of its parts? Multi-level issues in creativity and innovation. 2008.

[58] van Knippenberg D, Mell JN. Past, Present, and Potential Future of Team Diversity Research: From Compositional Diversity to Emergent Diversity. Organ Behav Hum Decis Process Behav Hum Decis Process. 2016;136: 135–145.

[59] De Dreu CKW, Nijstad BA, van Knippenberg D. Motivated information processing in group judgment and decision making. Personal Soc Psychol Rev. 2008; 22–49. 10.1177/1088868307304092 18453471

[60] Hinsz BV, Tindale SR, Vollrath AD. The emerging conceptualization of groups as information processors. Psychol Bull. 1997;121: 43–64. 10.1037/0033-2909.121.1.43 9000891

[61] Madrid HP, Totterdell P, Niven K, Barros E. Leader affective presence and innovation in teams. J Appl Psychol. 2015;101: 673–686. 10.1037/apl0000078 26783828

[62] De Dreu CKW. When Too Little or Too Much Hurts: Evidence for a Curvilinear Relationship Between Task Conflict and Innovation in Teams. J Manage. 2006;32: 83–107.

[63] Hoever IJ, Zhou J, Knippenberg DVAN. Different strokes for different teams: The contingent effects of positive and negative feedback on the creativity of informationally homogeneous and diverse teams. Acad Manag J. 2018;61: 2159–2181.

[64] Rietzschel EF, Nijstad BA, Stroebe W. Relative accessibility of domain knowledge and creativity: The effects of knowledge activation on the quantity and originality of generated ideas. J Exp Soc Psychol. 2007;43: 933–946.

[65] Gebert D, Boerner S, Kearney E. Fostering Team Innovation: Why Is It Important to Combine Opposing Action Strategies? Organ Sci. 2010;21: 593–608.

[66] Shin Y. Positive Group Affect and Team Creativity: Mediation of Team Reflexivity and Promotion Focus. Small Gr Res. 2014;45: 337–364.

[67] Somech A. The Effects of Leadership Style and Team Process on Performance and Innovation in Functionally Heterogeneous Teams. J Manage. 2006;32: 132–157.

[68] Weingart LR. How did they do that? The ways and means of studying group process. Res Organ Behav. 1997;19: 189–239.

[69] Paulus PB, Nijstad BA. Group Creativity: Innovation through Collaboration. Oxford University Press 2003.

[70] Lıcuanan BF, Dailey LR, Mumford MD. Idea evaluation: Error in evaluating highly original ideas. J Creat Behav. 2007;41: 1–27.

[71] Morrison EW, Vancouver JB. Within-person analysis of information seeking: The effects of perceived costs and benefits. J Manage. 2000;26: 119–137.

[72] Centola D, Macy M. Complex Contagions and the Weakness of Long Ties. Am J Sociol. 2007;113: 702–734. 10.1086/521848

[73] Kilduff M, Brass DJ. Organizational Social Network Research: Core Ideas and Key Debates. Acad Manag Ann. 2010;4: 317–357.

[74] Sparrowe RT, Liden RC, Wayne SJ, Kraimer ML. Social networks and the performance of individuals and groups. Acad Manag J. 2001;44: 316–325.

[75] Tost LP, Gino F, Larrick RP. Power, competitiveness, and advice taking: Why the powerful don’t listen. Organ Behav Hum Decis Process. 2012;117: 53–65.

[76] Mehra A, Kilduff M, Brass DJ. The Social Networks of High and Low Self-Monitors: Implications for Workplace Performance. Adm Sci Q. 2001;46: 121–146.

[77] Borgatti SP, Foster PC. The network paradigm in organization research: A review and typology. J Manage. 2003;29: 991–1013.

[78] Kilduff M, Krackhardt DJ. Interpersonal Networks in Organizations: Cognition, Personality, Dynamics, and Culture. Cambridge University Press 2008.

[79] Lee LL. Intra-Organizational Networks. IT Governance in a Networked World. Oxford, UK: IGI Global; 2011 pp. 155–176. 10.4018/978-1-60566-084-4.ch007

[80] Weitz BA, Bradford KD. Personal Selling and Sales Management: A Relationship Marketing Perspective. J Acad Mark Sci. 1999;27: 241–254.

[81] Ingram TN, Lee KS, Lucas GH. Commitment and involvement: Assessing a salesforce typology. J Acad Mark Sci. 1991;19: 187–197.

[82] Jones E, Brown SP, Zoltners AA, Weitz BA. The Changing Environment of Selling and Sales Management. J Pers Sell Sales Manag. 2005;25: 105–111.

[83] Dixon AL, Gassenheimer JB, Barr TF. Identifying the lone wolf: A team perspective. J Pers Sell Sales Manag. 2003;23: 205–219.

[84] Dietz B, van Knippenberg D, Hirst G. Outperforming whom? A multilevel study of performance-prove goal orientation, performance, and the moderating role of shared team identification. J Appl Psychol. 2015;100: 1811 10.1037/a0038888 26011723

[85] Gong Y, Huang JC, Farh J-L. Employee learning orientation, transformational leadership, and employee creativity: The mediating role of employee creative self-efficacy. Acad Manag J. 2009;52: 765–778.

[86] Alexander L, van Knippenberg D. Teams in pursuit of radical innovation: A Goal Orientation Perspective. Acad Manag Rev. 2014;39: 423–438.

[87] Litchfield RC. Brainstorming reconsidered: A goal-based view. Acad Manag Rev. 2008;33: 649–668.

[88] Oh H, Chung M-H, Labianca G. Group Social Capital and Group Effectiveness: The Role of Informal Socializing Ties. Acad Manag J. 2004;47: 860–875.

[89] Ancona DG, Caldwell DF. Bridging the Boundary: External Activity and Performance in Organizational Teams. Adm Sci Q. 1992 10.2307/2393475

[90] Gong Y, Wu J, Song LJ, Zhang Z. Dual tuning in creative processes: Joint contributions of intrinsic and extrinsic motivational orientations. J Appl Psychol. 2017 10.1037/apl0000185 28150986

[91] Farmer SM, Tierney P, Kung-Mcintyre K. Employee creativity in Taiwan: An application of role identity theory. Acad Manag J. 2003;46: 618–630.

[92] Zhou H, Long L-R. Effects of job insecurity and creative self-efficacy on employees’ creativity. Acta Psychol Sin. 2011;43: 929–940.

[93] LeBreton JM, Senter JL. Answers to 20 Questions About Interrater Reliability and Interrater Agreement. Organ Res Methods. 2007;11: 815–852.

[94] Borgatti SP, Everett MG, Johnson JC. Analyzing social networks. 2013.

[95] Perry-Smith JE. Social yet creative: The role of social relationships in facilitating individual creativity. Acad Manag J. 2006;49: 85–101.

[96] Zhou J, Shin SJ, Brass DJ, Choi J, Zhang Z-X. Social networks, personal values, and creativity: evidence for curvilinear and interaction effects. J Appl Psychol. 2009;94: 1544–1552. 10.1037/a0016285 19916661

[97] Bell ST, Villado AJ, Lukasik MA, Belau L, Briggs AL. Getting Specific about Demographic Diversity Variable and Team Performance Relationships: A Meta-Analysis. J Manage. 2011;37: 709–743.

[98] Hülsheger UR, Anderson NR, Salgado JF. Team-level predictors of innovation at work: a comprehensive meta-analysis spanning three decades of research. J Appl Psychol. 2009;94: 1128–1145. 10.1037/a0015978 19702361

[99] Long JA. jtools: Analysis and Presentation of Social Scientific Data. 2018.

[100] Anderson C, Willer R, Kilduff GJ, Brown CE. The origins of deference: When do people prefer lower status? J Pers Soc Psychol. 2012 10.1037/a0027409 22369047

[101] Bendersky C, Hays NA. Status Conflict in Groups. Organ Sci. 2011 10.1287/orsc.1110.0734

[102] De Dreu CKW, Weingart LR. Task versus relationship conflict, team performance, and team member satisfaction: A meta-analysis. J Appl Psychol. 2003. 10.1037/0021-9010.88.4.741 12940412

[103] Samba C, Van Knippenberg D, Miller CC. The impact of strategic dissent on organizational outcomes: A meta-analytic integration. Strateg Manag J. 2018 10.1002/smj.2710

[104] Leenders RTAJ, van Engelen JML, Kratzer J. Virtuality, communication, and new product team creativity: A social network perspective. J Eng Technol Manag. 2003;20: 69–92.

[105] Perry-Smith JE, Mannucci PV. From Creativity to Innovation: The Social Network Drivers of the Four Phases of the Idea Journey. Acad Manag Rev. 2015;42: 53–79.

[106] Yuan Y. The Emergence of Team Creativity: A Social Network Perspective In: Paulus PB, Nijstad BA, editors. The Oxford Handbook of Group Creativity and Innovation. Oxford University Press; 2019 pp. 131–154. 10.1093/oxfordhb/9780190648077.013.9

[107] Mueller J, Cronin MA. How relational processes support team creativity. Res Manag Groups Teams. 2009;12: 291–300. 10.1108/S1534-0856(2009)0000012014

[108] Kearney E, Gebert D, Voelpel SC. When and how diversity benefits teams: The importance of team members’ need for cognition. Acad Manag J. 2009;52: 581–598.

[109] Nederveen Pieterse A, van Knippenberg D, Van Dierendonck D, Pieterse AN, van Knippenberg D, Van Dierendonck D. Cultural Diversity and Team Performance: The Role of Team Member Goal Orientation. Acad Manag J. 2013;56: 782–804. 10.5465/amj.2010.0992

[110] Kozlowski SWJ, Klein KJ. A multilevel approach to theory and research in organizations: Contextual, temporal, and emergent processes. Multilevel theory Res methods Organ Found extensions new Dir. 2000; 3–90.

[111] Kozlowski SWJ, Bell BS. Work groups and teams in organizations. Handbook of Psychology. 2003 pp. 333–375.

[112] Barrick MR, Stewart GL, Neubert MJ, Mount MK. Relating member ability and personality to work-team processes and team effectiveness. J Appl Psychol. 1998;83: 377–391.

[113] Halfhill T, Sundstrom E, Lahner J, Calderone W, Nielsen TM. Group Personality Composition and Group Effectiveness: An Integrative Review of Empirical Research. Small Gr Res. 2005;36: 83–105.

[114] Humphrey SE, Hollenbeck JR, Meyer CJ, Ilgen DR. Personality Configurations in Self-Managed Teams: A Natural Experiment on the Effects of Maximizing and Minimizing Variance in Traits. J Appl Soc Psychol. 2011;41: 1701–1732. 10.1111/j.1559-1816.2011.00778.x

[115] Gully SM, Devine DJ, Whitney DJ. A Meta-Analysis of Cohesion and Performance: Effects of Level of Analysis and Task Interdependence. Small Gr Res. 1995;26: 497–520.

[116] Madjar N, Greenberg E, Chen Z. Factors for radical creativity, incremental creativity, and routine, noncreative performance. J Appl Psychol. 2011;96: 730–743. 10.1037/a0022416 21319879

[117] Alexiev AS, Jansen JJP, van den Bosch FAJ, Volberda HW. Top management team advice seeking and exploratory innovation: The moderating role of TMT heterogeneity. J Manag Stud. 2010;47: 1343–1364.

[118] Han JJJ, Han JJJ, Brass DJ. Human capital diversity in the creation of social capital for team creativity. J Organ Behav. 2014;35: 54–71.

[119] Ancona DG, Caldwell D. Demography and Design: Predictors of New Product Team Performance. Organ Sci. 1992;3: 321–341.

[120] Faul F, Erdfelder E, Lang AG, Buchner A. G * Power 3: A flexible statistical power analysis program for the social, behavioral, and biomedical sciences. Behav Res Methods. 2007;39: 175–191. 10.3758/bf03193146 17695343

[121] Ellis PD. The essential guide to effect sizes: Statistical power, meta-analysis, and the interpretation of research results. Cambridge University Press; 2010.

[122] Hirst G, Van Knippenberg D, Chen CH, Sacramento CA. How does bureaucracy impact individual creativity? A cross-level investigation of team contextual influences on goal orientation-creativity relationships. Acad Manag J. 2011;54: 624–641.

